# Leukocyte telomere length and bipolar disorder risk: evidence from Mendelian randomization analysis

**DOI:** 10.7717/peerj.15129

**Published:** 2023-03-31

**Authors:** Likui Lu, Hongtao Zeng, Bangbei Wan, Miao Sun

**Affiliations:** 1 The First Affiliated Hospital of Soochow University, Institute for Fetology, Suzhou, Jiangsu, China; 2 Hainan Women and Children’s Medical Center, Reproductive Medical Center, Haikou, Hainan, China; 3 Central South University Xiangya School of Medicine Affiliated Haikou Hospital, Department of Urology, Haikou, Hainan, China; 4 Dushu Lake Hospital Affiliated to Soochow University, Suzhou, Jiangsu Province, China

**Keywords:** Leukocyte telomere length, Bipolar disorder, Mendelian randomization, Genome-wide association study, Single-nucleotide polymorphisms, Spinal stenosis

## Abstract

**Objective:**

We aim to test whether leukocyte telomere length (LTL) is causally associated with the risk of bipolar disorder (BD) using the Mendelian randomization (MR) method.

**Methods:**

Results of a genome-wide association study (GWAS) conducted with 472,174 individuals of European descent were used to screen for single-nucleotide polymorphisms (SNPs) related with LTL traits. Summary-level data for BD (7,647 cases and 27,303 controls) were obtained from UK Biobank. An inverse-variance-weighted (IVW) method was employed as the primary MR analysis. Sensitivity analyses were conducted *via* MR-Egger, maximum likelihood, MR-pleiotropy residual sum outlier (MR-PRESSO), and MR-robust adjusted profile score (MR-RAPS) methods. Finally, the MR Steiger test was utilized to validate the hypothesized relationship between exposure and outcome.

**Results:**

Two-sample MR analysis revealed inverse relationships between genetically predicted LTL and BD risk (IVW OR [odds ratio] = 0.800, 95% CI [0.647–0.989] *P* = 0.039). Genetically predicted LTL exhibits a consistent connection with BD across five MR methods. Sensitivity analyses showed that the genetically determined effect of LTL on BD was stable and reliable. Furthermore, the MR Steiger test demonstrated that LTL was causal for BD rather than the opposite (*P* < 0.001).

**Conclusion:**

Our findings show that genetically determined LTL reduces the risk of BD. More research is required to clarify the mechanisms underlying this apparent causal connection. In addition, these findings may be useful for developing strategies for the prevention and treatment of BD.

## Introduction

Bipolar disorder (BD) is a severe neuropsychiatric condition characterized by recurrent periods of mania and depression that impair cognition, perception, emotion, and social interaction. The cause of BD is unknown, although genetic, neurochemical, and structural abnormalities, as well as stress, may contribute (McIntyre et al., 2020). BD is more prevalent in persons with a family history of BD and those suffering from depression, anxiety problems, or substance use issues (Bauer, 2022). BD is a significant public health issue and a large contributor to the global burden of illness due to its lifetime frequency of 1–2%, increased morbidity and mortality, beginning in young adulthood, and typically chronic course (Stahl et al., 2019). According to Fries et al. (2020), BD is a disease that accelerates aging in both clinical and molecular features. The causes of the illness and any associated effective therapy approaches are still unknown despite the considerable effort being put into examining the mechanisms underlying BD. Because of this, it is becoming more important to find people who are likely to get BD and could benefit from early preventive methods.

There is evidence that certain mental illnesses are linked to a faster rate of biological aging, either at the organismal or even the cellular level (Lindqvist et al., 2015). The telomere length (TL) is an emerging indicator of cellular aging that is frequently tested in leukocytes (as LTL). Telomeres are areas of repeating nucleotide sequences at the end of eukaryotic chromosomes that play a crucial function in chromosomal integrity. LTL is known as the ”molecular clock” related to the senescence of cells and organisms (Vaiserman & Krasnienkov, 2020). Epidemiological studies have shown evidence that shortened LTL is linked to several psychiatric disorders, including major depressive disorder (Monroy-Jaramillo, Dyukova & Walss-Bass, 2018; Pisanu et al., 2020a; Pisanu et al., 2020b; Wang et al., 2017), anxiety (Monroy-Jaramillo, Dyukova & Walss-Bass, 2018; Wang et al., 2017), schizophrenia (Ayora et al., 2022; Wolkowitz et al., 2017). The occurrence of BD may be closely related to these diseases, which may all be affected by shortened LTL. As a result, we speculate that the change of LTL may be closely related to the occurrence of BD. Observational study results indicate connections between LTL and BD (Ferensztajn-Rochowiak et al., 2021; Huang et al., 2018; Joo et al., 2021). Despite being helpful, these observational studies are vulnerable to confounding factors, which can lead to inaccurate causal conclusions (Grimes & Schulz, 2002). Hence, randomized controlled trials are required to demonstrate the validity of the relationships found in observational studies.

Randomized studies on LTL are challenging to conduct because of the need for large sample sizes and extensive follow-up periods. Because of this, establishing a causal link between LTL and diseases might be difficult. To establish whether the alleged connection between LTL and diseases is a causal one, an effective method must be identified. Mendelian randomization (MR) provides the opportunity to solve this issue explicitly (Do et al., 2013; Emdin, Khera & Kathiresan, 2017; Frikke-Schmidt et al., 2008; Voight et al., 2012). Since genetic mutations are intrinsic and unaffected by environmental circumstances, the MR research approach employing SNP as an instrumental variable can effectively control the interference of confounding variables, similar to a randomized controlled trial. In addition, genetic variation can have an effect on outcomes, but outcomes cannot have an impact on genes; hence, there is no possibility of inferring reverse causality. MR relies on the following assumptions: the genetic instrument should be highly related to the exposure but not confounders. The genetic variant should solely influence the outcome *via* the risk factor (Emdin, Khera & Kathiresan, 2017). Hence, establishing causality is possible with a genetic instrument that meets all MR assumptions.

Therefore, this study used a two-sample MR analysis and a variety of sensitivity analyses to investigate the causal link between LTL and BD risk. The discovered causal links between LTL and BD risk will help progress research into early preventive or diagnosis measures.

## Methods

### Overall study design

In this study, we utilized the two-sample MR method to evaluate the causal relationship of LTL with the risk of BD. The two-sample MR analyses used summary-level data from IEU Open GWAS (https://gwas.mrcieu.ac.uk/), namely LTL (472,174 individuals, ieu-b-4879) and BD (34,950 individuals, ebi-a-GCST003724). The relevant ethics board authorized the initial GWAS, and all participants provided informed consent.

### Assumptions of the Mendelian randomization study

**Figure 1:**
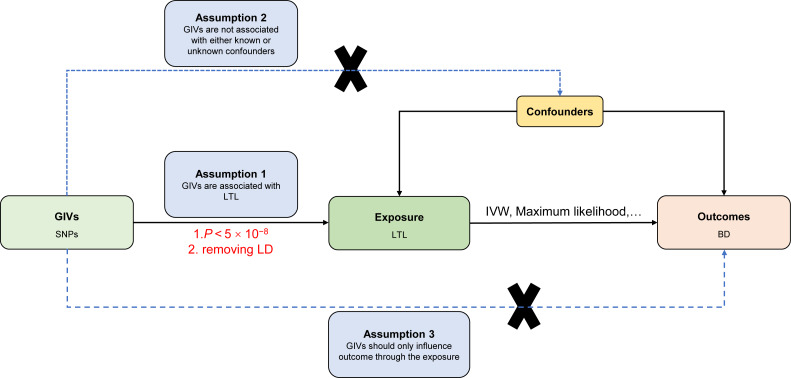
Directed acyclic graph of the MR framework investigating the causal relationship between LTL and BD. Instrumental variable assumptions: (1) Genetic instrument variables (GIVs) should be strongly associated with LTL. (2) The GIVs must not be associated with confounders that may play a role in the relationship between LTL and BD. (3) The GIVs should only influence the BD *via* LTL. SNPs, single-nucleotide polymorphisms; LTL, leukocyte telomere length; BD, bipolar disorder; IVW, inverse-variance-weighted; MR, Mendelian randomization.

The current MR study must acknowledge and accept these three key assumptions: (1) Genetic instrument variables (GIVs) should be strongly associated with LTL. (2) The GIVs must not be associated with confounders that may affect the relationship between LTL and BD. (3) The GIVs should only influence the BD *via* LTL (horizontal pleiotropy does not exist) (Davey Smith & Hemani, 2014; Smith & Ebrahim, 2003). Figure 1 illustrates the MR study’s assumptions and its overall design.

### Two-sample MR

#### SNP selection

After obtaining the GWAS summary-level data for LTL, we performed several quality control measures to decide which instrumental SNPs could be included in subsequent MR analyses. First, genome-wide significant SNPs associated with LTL (*P* < 5 × 10^−8^) were obtained. Second, it was critical to establish that none of the LTL-related instrumental SNPs were in linkage disequilibrium (LD). In this study, the LD between SNPs was determined by employing the clumping method (*r*^2^ < 0.001, window size = 10,000 kb) on European samples obtained from the 1000 Genomes Project. Among the pairs of SNPs where the level of LD *r*^2^ exceeds the stated threshold (*r*^2^ = 0.001), only the SNP with the lowest *P* value is retained. Moreover, the F statistic was calculated according to previous studies (Pierce, Ahsan & Vanderweele, 2011; Wu et al., 2020) to estimate the instrument strength. When the F statistic for the instrument-exposure correlation was significantly higher than 10, it indicated a low probability of weak instrumental variable bias (Davies, Holmes & Davey Smith , 2018).

### Primary analyses

The effect of LTL on BD risk was estimated using the inverse-variance-weighted (IVW) model, with LTL serving as the exposure and BD as the outcome. The IVW method combines Wald estimates for each SNP using a meta-analysis approach to determine the overall estimates of the impact of the exposure on the result (Burgess, Butterworth & Thompson, 2013).

#### Sensitivity analyses

To test the robustness of primary analysis, four methods were used, including MR-Egger (Bowden, Davey Smith & Burgess, 2015), Maximum likelihood (Xue, Shen & Pan, 2021), MR-pleiotropy residual sum outlier (MR-PRESSO) (Verbanck et al, 2018), and robust adjusted profile score (MR-RAPS) methods (Cui & Tian, 2021) to test the reliability and stability of the results. In short, MR-Egger recalculates the IVW causal estimates while taking the intercept out of the equation. The maximum likelihood approach assumes that each SNP’s effect on the outcome is the same; therefore, it may provide more robust results when the measurement error exists. MR-PRESSO is an approach for detecting and correcting outliers in linear IVW data. MR-RAPS accounts for the measurement error in SNP-exposure effects and is impartial in the presence of numerous weak instruments and robust to systematic and idiosyncratic pleiotropy. However, each of the above methods has some advantages and disadvantages. For example, the IVW method is the most powerful resilient approach when all variants are valid instrumental variables (IVs), as it is at its most effective when using only valid IVs. The efficiency of MR-Egger procedures is drastically lower (Burgess et al., 2020). In addition, to estimate consistently, the MR-Egger method requires the InSIDE (Instrument Strength Independent of Direct Effect) assumption. MR-RAPS works best when pleiotropic effects are actually normally distributed about zero. The MR-PRESSO is useful when there are few genetic variants with heterogeneous ratio estimates, but it is less valuable when there are many mildly pleiotropic variants or when the average pleiotropic effect of non-outliers is not zero. Due to variances in analysis platforms, experimental circumstances, inclusion populations, and SNPs, the assessment of causal effects may be impacted by heterogeneity in two-sample MR analyses. This study, therefore, examined the IVW and MR-Egger estimations for heterogeneity (Hemani et al., 2018). The heterogeneities were measured using the Cochran Q statistic; a *P* value (*P*-het) > 0.05 indicated no heterogeneity in the included instrumental variables. Hence the influence of heterogeneity on the assessment of causal effects could be disregarded. If there was heterogeneity, the random-effects model was employed to determine the effect size (Julian et al., 2021; Li et al., 2022). In addition, it is essential to determine if pleiotropy occurs in the MR causal inference. It is possible to evaluate pleiotropy using the Egger model’s intercept statistically; departures from 0 suggest the presence of directional pleiotropy (Burgess & Thompson, 2017). MR-PRESSO method was also employed to determine if the pleiotropy existed (Verbanck et al, 2018). Pleiotropy is unlikely in the causal analysis if *P* > 0.05. The MR Steiger test was performed to confirm the directionality of the exposure’s effect on the outcome, and *P* < 0.05 was considered statistically significant. Finally, according to a prior investigation (Brion, Shakhbazov & Visscher, 2013), the statistical power of our MR findings was assessed.

All MR studies were carried out in R (version 4.1.2; R Core Team, 2021) using the TwoSampleMR package (version 0.5.6) (Hemani et al., 2018).

## Results

All 133 distinct genetic variations connected to LTL were accessible in the summary statistics for BD. The F statistic for these SNPs exceeded 10 (range, 29.86–1628.82; mean, 119.94) for LTL, showing that there is little chance of weak-instrument bias (Fig. S1) (Baumeister et al., 2021). File S1 contains information on these SNPs in great detail.

**Figure 2:**
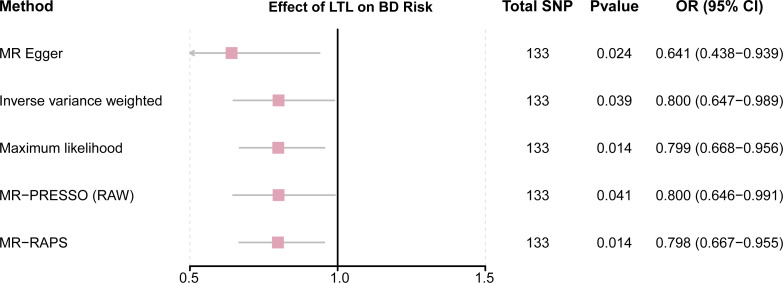
Forest plot to visualize causal effects of variation in LTL on BD. Presented odds ratios (OR) and confidence intervals (CI) correspond to the effects of LTL on BD. The results of MR analyses using various analysis methods (MR–Egger, maximum likelihood, MR-PRESSO, MR–RAPS, IVW) are presented for comparison. Total single-nucleotide polymorphism (SNP) indicates the number of genetic variants used as instruments for MR analysis.

**Figure 3:**
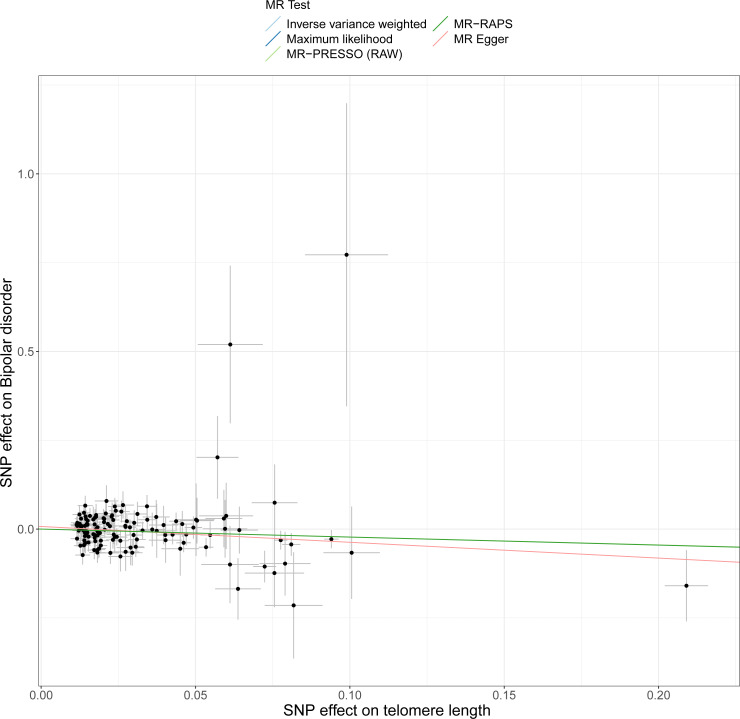
Scatter plots of LTL with the risk of BD. Scatter plot demonstrating the effect of each LTL-associated SNP on BD on the log-odds scale. The slopes of each line represent the causal association for each method.

**Figure 4:**
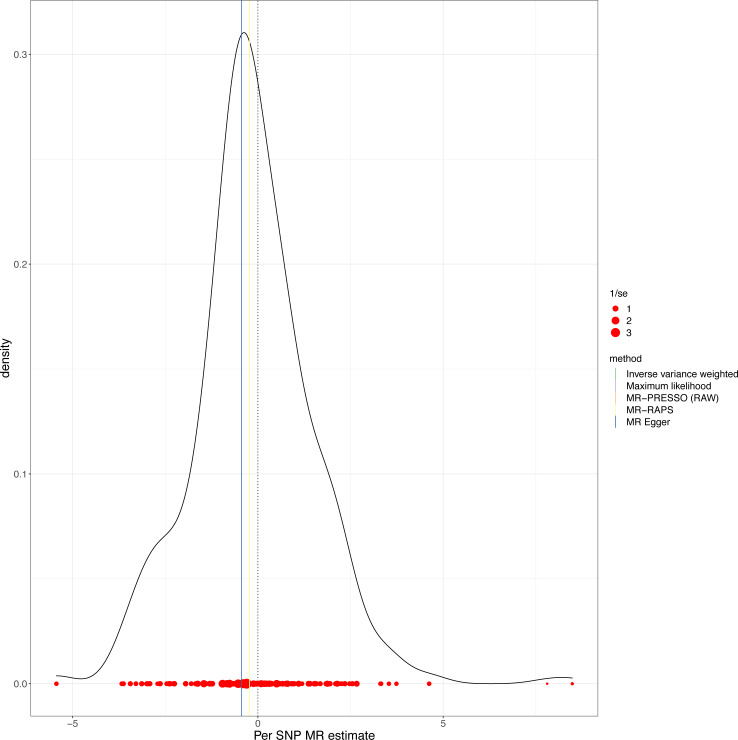
MR density plots to visualize the overall heterogeneity of MR estimates for the effect of LTL on BD. MR, Mendelian randomization; SNP, single-nucleotide polymorphism.

**Figure 5:**
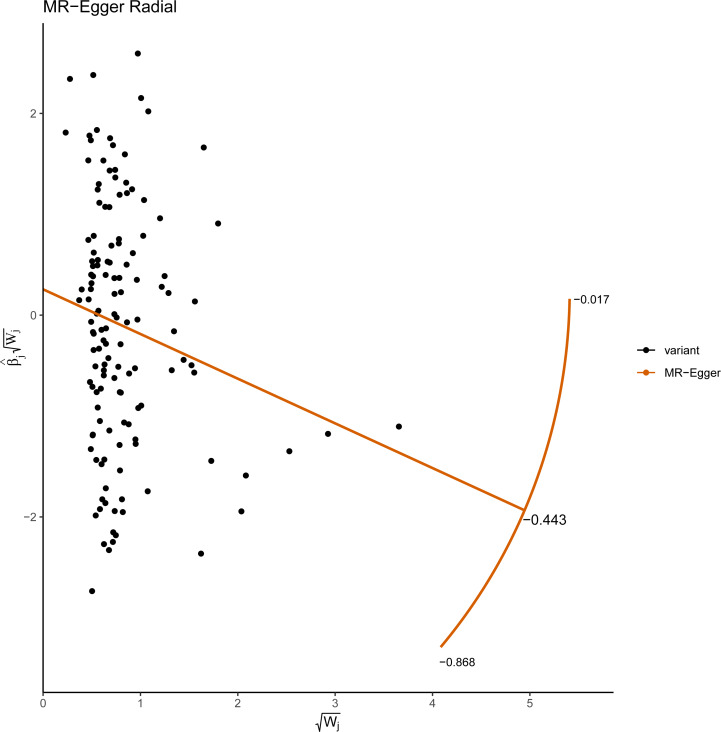
Radial plots of the MR-Egger test analyzed the outlier SNPs. The *X* axis represents the square root of the actual weight that every SNP gets in the IVW analysis. The *Y* axis represents each SNP’s ratio estimate multiplied by the same square-root weight.

This study demonstrated that genetically determined LTL has a negative association with BD risk; the OR was 0.800 (95% CI [0.647–0.989]; *P* = 0.039) in the IVW analysis (Table 1) (Fig. 2). Subsequent heterogeneity study results demonstrated substantial heterogeneity among the GIVs (*P*-het = 0.001), so we utilized the random-effects model to directly estimate the aforementioned MR effect size. Genetically predicted LTL showed a broadly consistent association with BD across the different MR methods (Table 1). The scatter plots also revealed that the slopes of the results among LTL and BD assessed by various methodologies are all negative, and the steady correlation pattern demonstrates that our study results are pretty dependable (Fig. 3). Additionally, density plots also show that the predicted effect values of most SNPs fall within a rather narrow range, indicating the absence of significant heterogeneity in our research (Fig. 4). The intercept term estimated from MR-Egger was centered at the origin (*P*-intercept = 0.173), indicating that the results were unaffected by the directional pleiotropy. No outlier SNP was identified that resulted in enhanced pleiotropy in the overall MR estimate by MR-PRESSO analysis and MR-Egger test (Fig. 5). Moreover, even though the SNPs explained 3.26% of the variance of LTL, there was 83.6% power to detect the causal association between LTL and BD. The MR Steiger test was used to validate the causal assumption of LTL and BD, and the results proved that LTL’s influence on BD was the proper causal direction (*P* < 0.001).

**Table 1:** MR results of LTL on risk of BD.

Exposure	Method	No. of SNPs	OR (95% CI)	*P*	*P*-het	*P*-intercept
	MR Egger	133	0.641 (0.438–0.939)	0.024	0.001584	0.173404
	IVW	133	0.800 (0.647–0.989)	0.039	0.001255	
LTL	Maximum likelihood	133	0.799 (0.668–0.956)	0.014		
	MR-PRESSO (RAW)	133	0.800 (0.646–0.991)	0.041		
	MR-RAPS	133	0.798 (0.667–0.955)	0.014		

**Notes.**

MR Mendelian randomization LTL leukocyte telomere length BD bipolar disorder IVW inverse variance weighted MR-PRESSO Mendelian randomization-pleiotropy residual sum outlier MR-RAPS MR-robust adjusted profile score OR odds ratio *P*-het *P* value for heterogeneity using Cochran Q test *P*-intercept *P* value for MR-Egger intercept SNP single-nucleotide polymorphism

## Discussion

In the present study, we estimated the causative influence of LTL on the risk of BD using the MR method. We observed that genetically determined LTL was negatively associated with BD.

Telomeres are nucleoprotein structures that are present at the ends of each chromosome arm and help to keep the genome stable. Gene abnormalities that are connected with telomere maintenance in humans have been linked to a variety of germline and somatic degenerative illnesses, including dyskeratosis congenital and ulcerative colitis (Calado et al., 2009). In addition, telomere dysfunction has become one of the molecular hallmarks of cellular aging (Lopez-Otin et al., 2013). Therefore, TL may be related to many aging-related diseases. For example, Haycock et al. (2014) found that LTL was linked to a lower risk of coronary heart disease, even when traditional vascular risk factors were taken into account. Pousa et al. (2021) reported an inverse association between TL and distress-related mental disorders (including traumatic stress disorder, anxiety disorder, and depression). Interestingly, Roberts et al. (2014) indicated that both long and short TL may play a role in the pathogenesis of amnestic mild cognitive impairment (aMCI) and may be markers of increased risk of aMCI. In addition, according to the findings of Jebaraj and colleagues, chronic lymphocytic leukemia is characterized by short telomeres, which are linked to a poor prognosis, genomic complexity, and clonal evolution (Jebaraj et al., 2019).

In addition to the results of observational studies, there are many studies using MR methods that also show a causal association between telomere length (TL) and many diseases. For example, a comprehensive MR analysis by The Telomeres Mendelian Randomization Collaboration (2017) showed the strongest positive association between genetically predicted longer TL and a variety of cancers. There were also many MR studies reporting the relationship between TL and neurological diseases, including amyotrophic lateral sclerosis (Xia et al., 2021), Alzheimer’s disease (Gao et al., 2019; Rodriguez-Fernandez et al., 2022; Yu et al., 2021), multiple sclerosis (Liao et al., 2022; Shu, Li & Zhu, 2022). But there are also some MR studies showing that telomere length is not associated with some neurological traits, such as depression (Wium-Andersen et al., 2017), and Parkinson’s disease (Chen & Zhan, 2021).

Currently, observational studies exist on the association of TL with BD. Rizzo and colleagues revealed evidence of accelerated aging in BD in the form of shorter telomeres (a marker of cellular aging) (Rizzo et al., 2013). In addition, Huang et al. (2018) found BD patients had shorter LTLs than controls. Ferensztajn-Rochowiak et al. (2021) also found that BD patients had significantly shorter TL compared with the control group. In addition to observational studies, an MR analysis enrolling 131 patients with BD and 336 controls conducted by Pisanu et al. (2020a) and Pisanu et al. (2020b) indicated that there was no association between genetically determined LTL and BD risk. Nevertheless, Pisanu’s study had a limited number of participants, which is a significant limitation that has an impact on the outcomes of the MR analysis (Davies, Holmes & Davey Smith , 2018). Besides this, to our knowledge, there were no large-sample MR analyses to explore the causal relationship between telomere length and BD. Consequently, it is crucial to reevaluate whether it will produce different conclusions if more extensive GWAS datasets become available. In line with previous studies, utilizing the MR analysis based on the largest LTL and BD-related datasets, we found a negative association between LTL and BD risk. At the same time, to ensure the reliability of the results, we performed a series of sensitivity analyses. The results show strong consistency of our findings across methods. Taken together, these data indicate that LTL is a substantial protective factor for BD. In addition, LTL may be an essential indicator for predicting BD risk.

MR studies must satisfy three basic assumptions (Davies, Holmes & Davey Smith, 2018). In this study, we evaluated the veracity of these assumptions in various methods. We primarily assessed the relevance assumption through *P*-value (*P* < 5 × 10^−8^) and LD analyses. In addition, we also use the F statistic to rule out the presence of weak instrumental variables. Horizontal pleiotropy cannot exist due to the nature of the exclusion restriction assumption. Hence, many sensitivity analyses were performed to examine the impact of horizontal pleiotropy on our MR study. The estimates between LTL and BD were generally consistent throughout IVW and sensitivity analysis, making the conclusion trustworthy. Finally, it is challenging to ensure that the independence assumption is not violated due to the presence of confounders that cannot be assessed or are unknown from prior knowledge. There is a possibility that the link between a GIV and an outcome is confounded in certain samples due to a hidden population structure (Davies, Holmes & Davey Smith , 2018). Consequently, the exposure and outcome datasets utilized in this investigation were all sourced from European populations, thereby avoiding the confounding effects of diverse populations on causal analyses. However, it remains to be determined whether additional confounding factors influence the association between LTL and BD risk.

Despite the fact that two-sample MR is an excellent method for making causal inferences among exposures and outcomes employing summary statistics, we should proceed with caution due to a number of limitations. First, our study was conducted using European populations, which limits its ability to be applied to a larger group. Second, it is also possible that additional factors, such as other disease states, confound our results, but it is hard to avoid. In addition, in our work, telomere length was detected in leucocytes, and whether it also be able actually to reflect the telomere length of other organ tissues is unclear. Lastly, even though that a number of sensitivity analyses were done to investigate violations of exchangeability and exclusion limitation criteria, those assumptions remain unverifiable.

The large sample size is one of the strengths of this study. In addition, to our knowledge, no MR evaluating the link between LTL and BD has been done. MR research had advantages over conventional observational studies, such as the reduction of residual confounding risk. In addition, we performed a series of sensitivity assessments to verify the stability and reliability of the MR analysis results. As a consequence, we were able to provide novel insights that may help clarify the role of LTL in BD occurrence.

In summary, using the MR method, we found that LTL was a negative causal factor for BD risk. More research is required to establish how this potential cause-and-effect relationship works. Clinically, BD may be predicted by detecting LTL. In addition, because genetic variants cause the effect of LTL on BD is lifelong. Therefore, our findings are helpful in developing strategies for treating BD.

##  Supplemental Information

10.7717/peerj.15129/supp-1 Supplemental Information 1 F statistic of included SNPs associated with LTL in MR analysis The F statistic of these SNPs was greater than 10 (range, 29.86–1628.82; mean, 119.94) for LTL.

10.7717/peerj.15129/supp-2 Supplemental Information 2 Raw data and R code

10.7717/peerj.15129/supp-3 Supplemental Information 3 SNPs associated with leukocyte telomere length

10.7717/peerj.15129/supp-4 Supplemental Information 4 STROBE MR checklist

10.7717/peerj.15129/supp-5 Supplemental Information 5 Mendelian randomization study of the association between telomere length and bipolar disorder All 133 distinct genetic variations connected to telomere length were accessible in the summary statistics for bipolar disorder. Further Mendelian randomization analysis demonstrated that genetically determined telomere length has an inverse relationship with bipolar disorder. Created with Biorender.com.

## Abbreviations

LTL: leukocyte telomere length
BD: bipolar disorder
MR: mendelian randomization
SNPs: single-nucleotide polymorphisms
GWAS: genome-wide association study
IVW: inverse-variance-weighted
MR-PRESSO: Mendelian randomization-pleiotropy residual sum outlier
MR-RAPS: MR-robust adjusted profile score
OR: odds ratio
GIVs: genetic instrument variables
LD: linkage disequilibrium
aMCI: amnestic mild cognitive impairment

## Additional Information and Declarations

### Data Availability

The following information was supplied regarding data availability: The raw data is available in the Supplemental Files.
