# Supplementary figures and images for: Leukocyte telomere length and bipolar disorder risk: evidence from Mendelian randomization analysis
Source: PeerJ. 2023 Mar 31;11:e15129. doi: 10.7717/peerj.15129 (PMC10069421; doi:10.7717/peerj.15129)

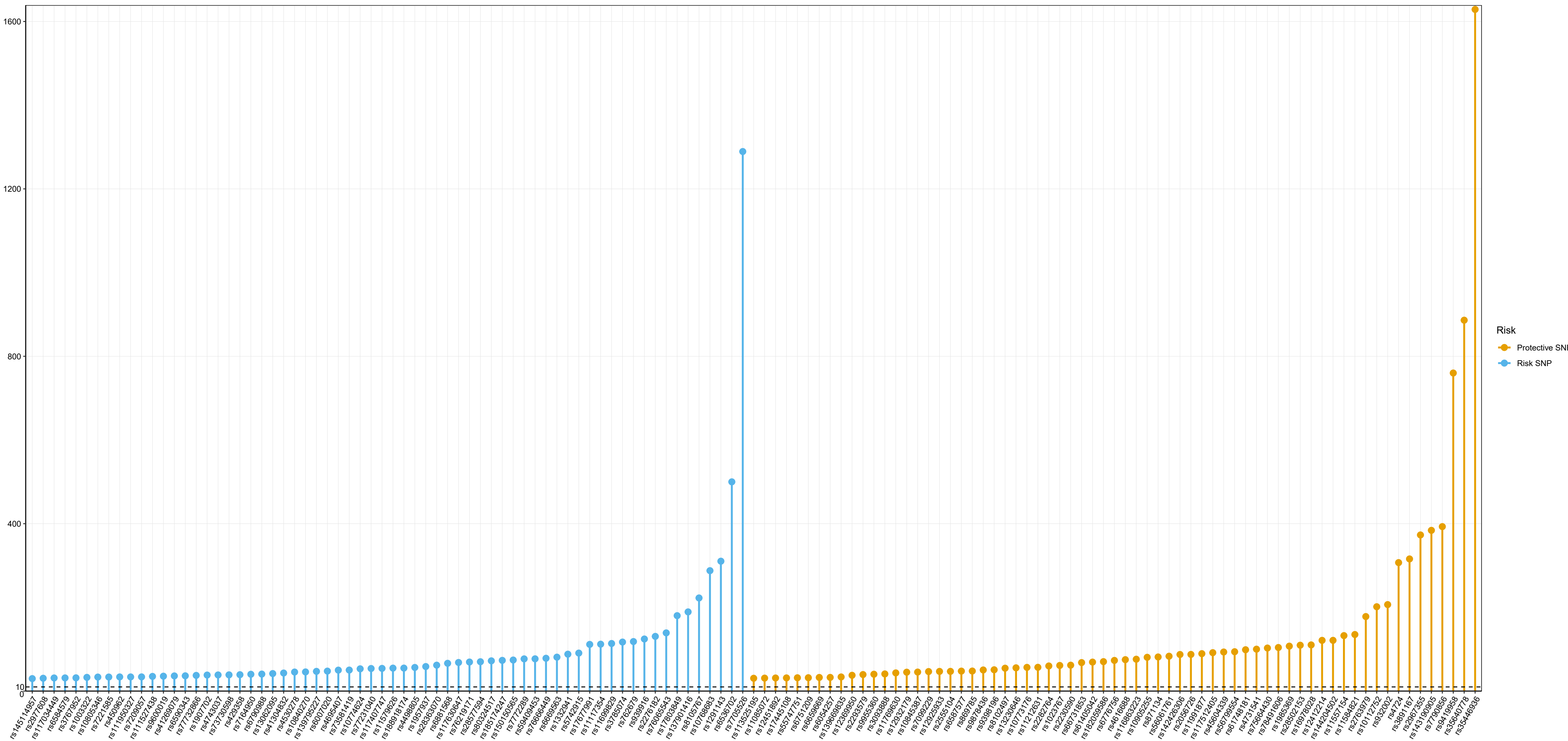

Supplement: Supplemental Information 1 — The F statistic of these SNPs was greater than 10 (range, 29.86–1628.82; mean, 119.94) for LTL. [file peerj-11-15129-s001.pdf]
