# Supplementary material for: Leukocyte telomere length and bipolar disorder risk: evidence from Mendelian randomization analysis
Source: PeerJ. 2023 Mar 31;11:e15129. doi: 10.7717/peerj.15129 (PMC10069421; doi:10.7717/peerj.15129)

Mendelian randomization study of the association between telomere length and bipolar disorder

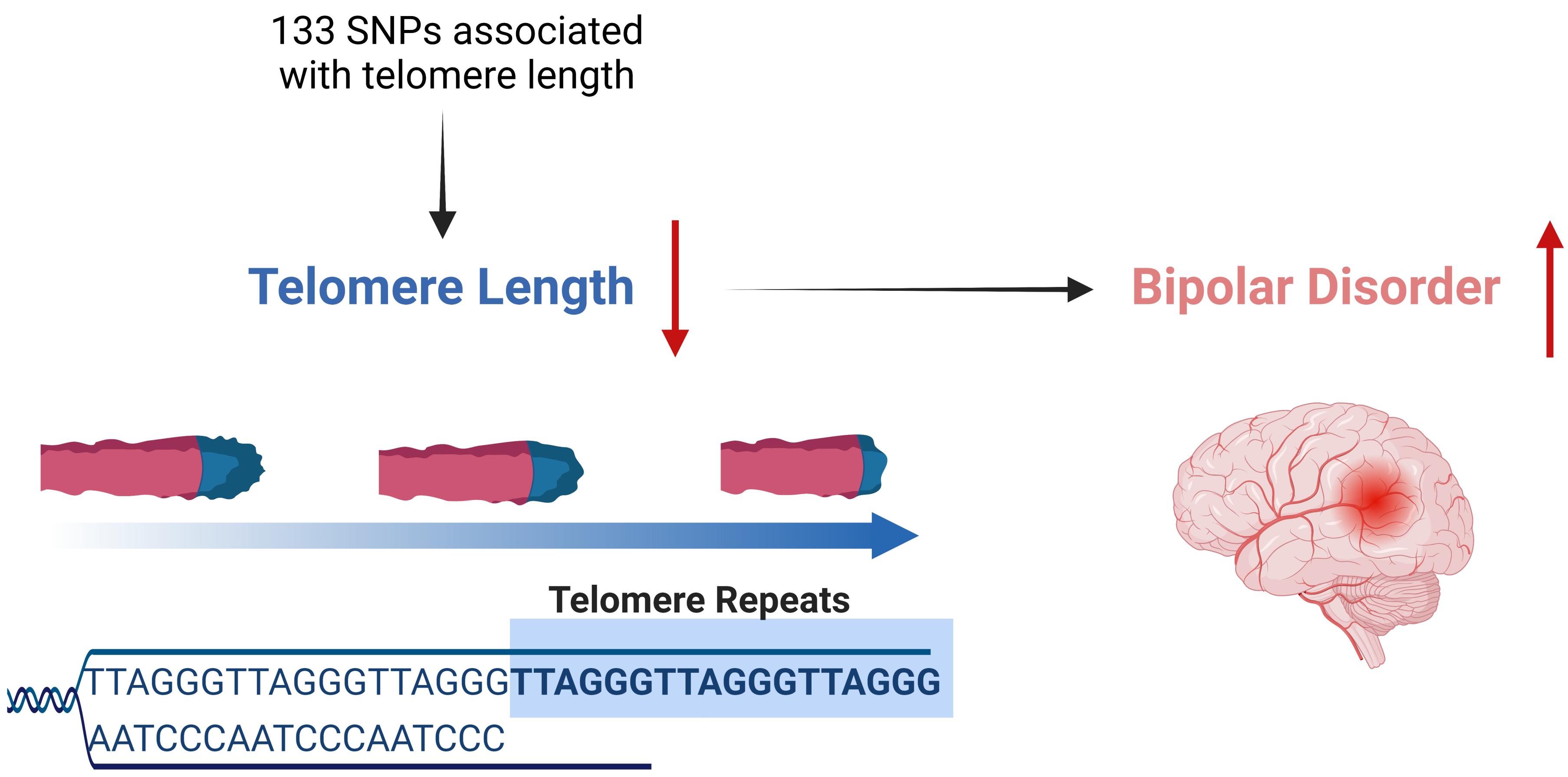

Supplement: Supplemental Information 5 — All 133 distinct genetic variations connected to telomere length were accessible in the summary statistics for bipolar disorder. Further Mendelian randomization analysis demonstrated that genetically determined telomere length has an inverse relationship with bipolar disorder. Created with Biorender.com. [file peerj-11-15129-s005.pdf]
